# Neurotransmitters and Sodium Channelopathies; Possible Link?

**DOI:** 10.15844/pedneurbriefs-31-3-1

**Published:** 2017-11

**Authors:** Michael F. Hammer, Alejandra D.C. Encinas

**Affiliations:** 1 University of Arizona Genetic Core, University of Arizona, Tucson, AZ; 2 Graduate Interdisciplinary Program in Genetics, University of Arizona, Tucson, AZ

**Keywords:** Seizures, Channelopathy, *SCN2A*, *SCN8A*, Nav1.2, Nav1.6, Dopamine, Serotonin, Therapy

## Abstract

Investigators from the University of British Columbia, Great Ormond Street Hospital for Children, and the National Hospital reported their findings on neurotransmitter deficiencies in two patients with mutations in voltage-gated sodium genes (*SCN2A* and *SCN8A*) discovered by whole exome sequencing.

Investigators from the University of British Columbia, Great Ormond Street Hospital for Children, and the National Hospital reported their findings on neurotransmitter deficiencies in two patients with mutations in voltage-gated sodium genes (*SCN2A* and *SCN8A*) discovered by whole exome sequencing.

In the first patient a de novo *SCN2A* splice-site mutation (c.2379+1G>A; p.Glu717Glyfs*30) that ultimately causes a premature stop at amino acid position 717 was discovered in a 10-year old male with epilepsy, cerebral/cerebellar atrophy, autism, and global developmental delays. Cerebrospinal fluid (CSF) analysis showed low neurotransmitter levels of homovanillic acid (HVA), 5-hydroxyindoleacetic acid (5-HIAA), neopterin, and tetrahydrobiopterin at 3.7 years; decreased 5-HIAA and HVA levels at 7.5 years, and a slightly lowered HVA level (217; 329-852nmol/L) at 9.6 years. Two separate oral treatments of 5-hydroxytrptophan (5HTP) and L-Dopa/Carbidopa were administered, the first began at 4-years and was stopped after 4 months after a reported decrease in seizure frequency with no improvement in speech and language development. At 7.7-years 5HTP and L-Dopa/Carbidopa were given leading to a 5-month seizure free period after which seizures recurred. A dopa-agonist, pramipexole (0.625 mg TID), was then introduced with a decreased L-Dopa/Carbidopa dosage and no change to 5HTP with seizure frequency dropping to once every 3-4 weeks.

The second case involved a 9-year old female with a de novo *SCN8A* missense mutation (c.5615G>A; p.Arg1872Gln) that presented with early onset drug-resistant epilepsy. CSF analysis revealed decreased 5-methylterahydrofolate (5-MTHF) neurotransmitter level at 6-years; and decreased levels of HVA, 5-HIAA, and 5-MTHF at 8-years. A trial of oral folinic acid (10mg/day) did not lead to clinical improvement and 5HTP and L-Dopa/Carbidopa treatment are being considered. [1]

COMMENTARY. The authors hypothesize that the loss-of-function mutation in the Nav1.2 channel and the missense mutation in the C-terminal of the Nav1.6 channel are causally linked to decreased vesicular biogenic amine synaptic release, possibly via increased cellular pools of Ca2+. Loss of function in the case of the *SCN2A* mutation could lead to activation of the Na+/Ca2+ exchanger at the synaptic junction, increasing the net intracellular Ca2+, with consequences for neurotransmitter release from the vesicles. The loss of the positively charged arginine at 1872 in *SCN8A* has been predicted to increase the number of channels that reopen during sustained depolarization in the neuron, leading to heightened channel activity and epileptogenic persistent current [2]. This C-terminus missense mutation is located in a region of the channel that is involved in the interaction with accessory proteins (e.g., calmodulin) that regulate neurotransmitter release.

The suggestion that the downstream effects of these mutations may ultimately explain specific symptoms of these channelopathies such as ataxia in case 1 and developmental regression with impairment of gross and fine motor skills and dyskinesia in case 2, leads to a set of testable hypotheses. With the increasing numbers of drug-resistant epilepsies resulting from *SCN2A* and *SCN8A* mutations being reported, there is increasing opportunity to gather the necessary data to investigate the potential link between neurotransmitters and voltage-gated sodium channelopathies. Given the semi-invasive nature of CSF testing it may be challenging to obtain longitudinal data for neurotransmitter levels. Other challenges include controlling for the known variable clinical symptoms associated with different patients and mutations, as well as documenting the many changes in treatment and medication regimen that are typically required to keep seizures as well controlled as possible.
